# Neonatal diabetes mellitus due to pancreatic agenesis and pervasive developmental disorder

**DOI:** 10.1186/1824-7288-35-23

**Published:** 2009-07-31

**Authors:** Alessandro Giannattasio, Maria Pintaudi, Maria Margherita Mancardi, Francesca Maria Battaglia, Edvige Veneselli, Renata Lorini, Giuseppe d'Annunzio

**Affiliations:** 1 Department of Pediatrics, University of Genova, Giannina Gaslini Institute, Genova, Italy; 2 O.U. of Child Neuropsychiatry, Giannina Gaslini Institute, Genova, Italy

## Abstract

Recent studies suggested a link between type 1 diabetes mellitus and pervasive developmental disorder. Moreover, permanent neonatal diabetes mellitus due to pancreatic agenesis can be associated with neurological deficit involving cerebellar functions, but no association with pervasive developmental disorder has been described so far. Clinical and neuropsychological evaluation of a child with pancreatic agenesis, mental retardation and pervasive developmental disorder is reported.

## 

Dear Editor,

Recent studies suggested a link between type 1 diabetes mellitus and pervasive developmental disorder (PDD) and hypothesized a common autoimmune pathogenesis or shared genetic factors [1]. Confirmation of an association between PDD and type 1 diabetes mellitus is still lacking. On the contrary permanent neonatal diabetes mellitus (PNDM) due to pancreas agenesis is an extremely rare condition, associated with intra-uterine growth retardation (IUGR), cardiac defects, gall bladder or cerebellum agenesis [2]. No association with psychiatric symptoms has been described up to now. We describe a patient with association of PNDM due to pancreatic agenesis, mild mental retardation and PDD. The patient, a boy who is currently aged 6, is the first child of healthy unrelated parents (father from Italy and mother from Spain). He was born at 35 weeks of gestation, with severe IUGR (weight 1620 g, <3^rd ^percentile and length 45 cm, <3^rd ^percentile) [3]. In the first day of life he developed hyperglycemia: neonatal diabetes was diagnosed and treated with insulin, initially intravenously and then subcutaneously. Atrial septal defect was discovered, in absence of other dysmorphic features. Pancreas agenesis was detected by ultrasound, and then confirmed by magnetic resonance imaging (MRI). Pancreatic exocrine insufficiency was treated with enzymes. Glycemic control was characterized during the first two years of life by fluctuations despite strict dietary regimen and frequent variation of insulin dosages. When 3 month-old the child presented a severe episode of hypoglicemia (plasma glucose 30 mg%); intramuscular glucagon was administrated and no neurological damage was evident at subsequent neuroimaging studies. Karyotype was normal and search for Fragile-X Syndrome was negative, as well as genetic testing for the most common forms of neonatal diabetes [4]. Brain MRI and neurophysiologic evaluations (auditory brainstem evoked potential, nerve conduction velocities and electroencephalogram) were normal. Because of developmental delay with behavioural abnormalities (impaired language functions with echolalia, isolation, stereotypies, no emotional sharing, hyperactivity) he underwent neuropsychiatric evaluation when 4 year-old. No focal signs were observed at neurological examination. Global motricity and coordination resulted normal. Griffiths' mental developmental scales revealed mild mental retardation, with global developmental quotient of 70. Neuropsychological testing with Childhood Autism Rating Scale (CARS), Autism Diagnostic Interview (ADI) and Autism Diagnostic Observation Schedule (ADOS) lead to diagnosis of Pervasive Developmental Disorder Non Otherwise Specified (PDD-NOS), according to Diagnostic and Statistical Manual of Mental Disorders IV-R (DSM IV-R) criteria [5]. This is the first case of PDD diagnosed in a child with pancreas agenesis. Four patients from two consanguineous Pakistan families with pancreatic agenesis associated with cerebellar agenesis have been reported, so that association of both pancreatic system and central nervous system developmental failure has been previously described [6]. Cerebellum is considered to have an important role in cognitive network as well as in the development of PDD [7]; our patient might have had abnormalities of cerebellar development not detected by standard neuroimaging but sufficient to impair cognition and behaviour. Nevertheless, we cannot exclude that in our case other perinatal risk factors for PDD should have influenced the behavioural disorder, as reported for IUGR and congenital malformations [8]. We want to emphasize the potential association between PNDM due to pancreatic agenesis and PDD, for prompt identification and appropriate care. A possible syndromic association needs further cases to be established. The existence of a link between PNDM and PDD may provide important insights into the pathogenesis of both conditions.

## Competing interests

The authors declare that they have no competing interests.

## Authors' contributions

All Authors participated in the data collection and read and approved the final manuscript. AG and GdA conceived of the study, and participated in its design and coordination, MP and MMM partecipated in clinical evaluation, FMB stated neuropsychological analysis, EV and RL partecipated in writing this manuscript.
